# A handheld device for intra-cavity and ex vivo fluorescence imaging of breast conserving surgery margins with 5-aminolevulinic acid

**DOI:** 10.1186/s42490-024-00079-9

**Published:** 2024-06-01

**Authors:** Christopher Gibson, Shirley C. Wang, Arcturus Phoon, Nayana Thalanki Anantha, Kathryn Ottolino-Perry, Stephen Petropoulos, Zuha Qureshi, Vasanth Subramanian, Anam Shahid, Cristiana O’Brien, Steven Carcone, Suzanne Chung, Teresa Tsui, Viktor Son, Mayleen Sukhram, Fannong Meng, Susan J. Done, Alexandra M. Easson, Tulin Cil, Michael Reedijk, Wey L. Leong, Ralph S. DaCosta

**Affiliations:** 1 Princess Margaret Cancer Centre, University Health Network, 101 College Street, M5G 1L7 Toronto, Canada; 2 Department of Medical Biophysics, University of Toronto, 101 College Street, M5G 1L7 Toronto, Canada; 3 The Toronto Health Economics and Technology Assessment (THETA) Collaborative, University Health Network, 200 Elizabeth Street, 10th Floor Eaton Wing, M5G 2C4 Toronto, Canada; 4 Laboratory Medicine Program, University Health Network, 200 Elizabeth Street, 11th Floor Eaton Wing, M5G 2C4 Toronto, Canada; 5 Department of Laboratory Medicine and Pathobiology, Faculty of Medicine, University of Toronto, 1 King’s College Circle, M5S 1A8 Toronto, Canada; 6 Surgical Oncology Department, Princess Margaret Cancer Centre, University Health Network, 610 University Ave, M5T 2M9 Toronto, Canada; 7 Techna Institute, University Health Network, 124-100 College Street, M5G 1P5 Toronto, Canada

**Keywords:** Breast conserving surgery, Fluorescence imaging, Margin assessment

## Abstract

**Background:**

Visualization of cancer during breast conserving surgery (BCS) remains challenging; the BCS reoperation rate is reported to be 20-70% of patients. An urgent clinical need exists for real-time intraoperative visualization of breast carcinomas during BCS. We previously demonstrated the ability of a prototype imaging device to identify breast carcinoma in excised surgical specimens following 5-aminolevulinic acid (5-ALA) administration. However, this prototype device was not designed to image the surgical cavity for remaining carcinoma after the excised lumpectomy specimen is removed. A new handheld fluorescence (FL) imaging prototype device, designed to image both excised specimens and within the surgical cavity, was assessed in a clinical trial to evaluate its clinical utility for first-in-human, real-time intraoperative imaging during index BCS.

**Results:**

The imaging device combines consumer-grade imaging sensory technology with miniature light-emitting diodes (LEDs) and multiband optical filtering to capture high-resolution white light (WL) and FL digital images and videos. The technology allows for visualization of protoporphyrin IX (PpIX), which fluoresces red when excited by violet-blue light. To date, $n = 17$ patients have received $20\frac{\text {mg}}{\textrm{kg}}$ bodyweight (BW) 5-ALA orally 2-4 h before imaging to facilitate the accumulation of PpIX within tumour cells. Tissue types were identified based on their colour appearance. Breast tumours in sectioned lumpectomies appeared red, which contrasted against the green connective tissues and orange-brown adipose tissues. In addition, ductal carcinoma *in situ* (DCIS) that was missed during intraoperative standard of care was identified at the surgical margin at <1 mm depth. In addition, artifacts due to the surgical drape, illumination, and blood within the surgical cavity were discovered.

**Conclusions:**

This study has demonstrated the detection of a grossly occult positive margin intraoperatively. Artifacts from imaging within the surgical cavity have been identified, and potential mitigations have been proposed.

**Trial Registration:**

ClinicalTrials.gov Identifier: NCT01837225 (Trial start date is September 2010. It was registered to ClinicalTrials.gov retrospectively on April 23, 2013, then later updated on April 9, 2020, to reflect the introduction of the new imaging device.)

## Background

Visualization of cancer during breast conserving surgery (BCS) remains a challenge. Under standard white light (WL) operating room conditions, cancer-to-normal tissue contrast is low with naked-eye visualization and palpation. This limitation results in positive margins and a high BCS reoperation rate, reported to be 20-$70\%$ of patients [1–10]. Therefore, reducing positive margin rates is an essential goal in BCS [11]. There is an urgent need for real-time BCS imaging to assess the surgical cavity and excised specimens for breast carcinoma at the margins. We previously tested a proof-of-concept solution using a prototype imaging device called PRODIGI [12] and demonstrated its ability to identify breast carcinoma, specifically invasive ductal carcinoma and ductal carcinoma in situ, in lumpectomies following 5-aminolevulinic acid (5-ALA) administration. However, this prototype device was not designed to image within the surgical cavity, the optics were not optimized, and it was constructed primitively using consumer electronics.

As a next step, we, in collaboration with MolecuLight Inc. (Toronto, Canada), developed a new handheld fluorescence (FL) imaging prototype device (called Eagle) for real-time intraoperative FL imaging of the surgical cavity and excised breast specimens. The FL image-guidance technology is clinically safe [13–15] and offers an alternative, practical, cost-sensitive intraoperative imaging technology for surgeons and pathologists to naturally move the device freely over all areas to be assessed and visualize occult malignancy in surgical cavities during the index BCS procedure and in excised specimens. Other FL imaging instruments are commercially available, but they involve large, costly, cart-based systems [16] that are impractical and often infeasible options for BCS facilities. In addition, they do not fulfill the surgeon’s need to visualize both the surgical cavity and the excised surgical specimen.

The Eagle device combines an image sensor with miniature light-emitting diodes (LEDs) and multiband optical filtering to capture high-resolution colour WL and FL digital images and videos. The technology allows for imaging of porphyrins, including protoporphyrin IX (PpIX), which fluoresces red (peak emission at 635 nm wavelength) when excited by violet-blue light (approximately 400-410 nm) [17, 18]. PpIX is a metabolite of the prodrug 5-ALA, an endogenous non-protein amino acid that is part of heme biosynthesis in mammalian cells [19]. When delivered systemically, 5-ALA is taken by cells throughout the body and converted into heme. In cancer cells, defects in heme biosynthesis cause accumulation of PpIX [18, 20–25], which enables real-time visualization. 5-ALA has been used clinically in photodynamic diagnosis and therapy of premalignant and malignant disease. Its safety and clinical utility have been demonstrated in large clinical trials for FL image-guided surgery for malignant glioma [26–29]. As a result, oral 5-ALA hydrochloride (HCl) has been approved for FL image-guided surgery of high-grade glioma in approximately forty countries, including the USA [30].

Other clinical studies support the use of 5-ALA for the visualization of malignant tissue in the bladder [31], gastrointestinal tract [32], oral cavity [33, 34], lung [35, 36], and female genital tract [37–39]. We previously presented the results of a single-centre non-interventional Phase II randomized controlled trial (RCT) designed to characterize the imaging performance of the PRODIGI imaging device with two doses (15 and $30\frac{\text {mg}}{\textrm{kg}}$ bodyweight (BW)) of 5-ALA HCl versus no tumour contrast in patients with invasive breast cancer undergoing lumpectomy or mastectomy [12]. In this manuscript, the findings of a clinical trial are presented, whereby intraoperative imaging during BCS using our latest handheld FL imaging device after administration of $20\frac{\text {mg}}{\textrm{kg}}$ BW of 5-ALA HCl was performed. The $20\frac{\text {mg}}{\textrm{kg}}$ dose of 5-ALA HCl was selected based on the same FDA-approved dose for glioma [30]. The overall objective of this study is to evaluate the feasibility and estimate the diagnostic accuracy of the Eagle device and 5-ALA HCl ($20\frac{\text {mg}}{\textrm{kg}}$ BW) to visualize carcinoma in patients with carcinoma of the breast undergoing BCS. The objective of this manuscript is to present initial imaging results and the strengths and limitations of the imaging device in terms of its ability to visualize and distinguish breast tissues in the clinical setting (including within the surgical cavity) following administration of $20\frac{\text {mg}}{\textrm{kg}}$ BW of 5-ALA HCl. Furthermore, understanding the sources of the limitations of this approach and potential solutions was a secondary objective of this manuscript.

### Previous work: PRODIGI prototype

To assess the utility of FL-guided margin assessment intraoperatively during BCS following administration of 5-ALA, the PRODIGI prototype device was developed and introduced into an RCT [12] at the Princess Margaret Cancer Centre; Toronto, Canada (PMCC), $n=45$.

The PRODIGI device [12] included the components necessary to image PpIX: an excitation source, which was two 405 nm LEDs (Osram Sylvania, Wilmington, MA, USA), an imaging filter (Chroma Technology Corp, Bellows Falls, VT, USA) with green and red passbands, and a detector and display (both of which were provided by a consumer-grade point-and-shoot digital camera (Sony, Tokyo, Japan) capable of capturing 12 MP images and 720p video, with an integrated $3.5^{\prime \prime }$ touchscreen liquid crystal display (LCD)).

#### Limitations of the PRODIGI prototype

The PRODIGI device design was focused on imaging ex vivo specimens. However, PRODIGI had a bulky form factor (${30}\times {14}\times {10}$ cm, mass ${584}\,$g) and therefore, was not designed to fit within the surgical cavity to identify any remaining carcinoma left after the lumpectomy specimen was excised. In addition, the illumination was not optimal for imaging within an enclosed space, such as the surgical cavity. While healthy tissue FL was observed in the cavity, no intra-cavity areas of positive PpIX FL were identified [12]. Furthermore, the drape material which was a transparent plastic sheath secured to PRODIGI with sterile magnets, did not remain taut across the image sensor, resulting in blur and noise artifacts. During image capture, the long exposure (1-5 s) within the darkened room resulted in frequent blurry images due to motion.

Finally, clinical translation of an imaging platform that incorporates a consumer product is infeasible due to planned obsolescence and high cost with unnecessary features. Using knowledge gained in this clinical study, a new imaging platform was built to improve the clinical feasibility of 5-ALA-induced FL imaging to guide BCS margin assessment.

## Methods

### Device development requirements

To develop a new imaging device to detect 5-ALA-induced PpIX during BCS, there are several user-, regulatory-, and feasibility-motivated requirements necessary to inform the device design. Such user- and regulatory-motivated requirements aim to ensure that the device can address the clinical need and optimize usability. The technical specifications ensure that the device is designed and built to perform to a consistent standard.

#### Clinical usability

The new imaging device is intended to be used during standard-of-care cancer surgery to visualize carcinoma at surgical margins. It may be used to assess the margins of surgical cavities and lumpectomy specimens during BCS and in the pathology suite to evaluate the bulk tumour fluorescence on sectioned lumpectomy specimens. To be practical, the device must be able to be used in the operating room (OR) and pathology suite without interfering with surgical tools (e.g. titanium retractors, electrocautery), implanted devices (e.g. pacemakers, magnetic tracers), and the clinical environment. To minimize disruptions to the existing clinical workflow, scanning the surgical cavity should take no more than one minute—approximately 10 s per each of the six cavity surfaces (superior, inferior, medial, lateral, posterior, anterior). This does not include the extra time taken to manipulate the cavity to ensure that the best angle of each surface is facing the camera. Cavity manipulations may add up to an additional 10 s per surface, or about 1 min per cavity scan.

##### Form factor and ergonomics

ORs are increasingly filled with large, cart-based instruments that take up valuable space. Therefore, the device should be fully handheld and wireless, rather than tethered to an external computer or display. Wireless operation improves the ease of device rotation by the user without intrusion from a cable. As a result, the device will need to be battery-powered.

The device’s users within the OR include surgeons, surgical fellows, and nurses (scrub, circulating) for setting up the device and imaging. Outside the OR, primary users include pathologists and pathologists’ assistants (PAs). Consequently, the device will need to be operable with medical gloves, including double-gloved users whose gloves are covered with tissue, blood, or other debris, and with any standard glove material, including nitrile, latex, vinyl, and neoprene.

To ensure suitability for BCS, the device must be sized (${17}\times {13}\times {12}$ cm, mass ${557}\,$g) to image all internal surfaces of surgical cavities. Therefore, the imaging components must be able to navigate a surgical cavity with a narrow opening and curved, textured walls. In some cases, surgical cavity incisions may be made on the lateral side of the breast and “tunnelled” to the tumour. In this case, a device with a long shaft capable of imaging deep, narrow cavities is required. The device must also be capable of imaging all exterior surfaces of the lumpectomy specimen and additional margin shaves (if applicable) excised from the cavity. Post hoc analysis of data collected in the PRODIGI study revealed that lumpectomies were approximately ellipsoidal with mean axis lengths $4.0\pm {1.6}\,\text {cm}\times 6.5\pm {2.4}\,\text {cm}\times 6.9\pm {3.0}\,\text {cm}$ ($n=34$, mean ± standard deviation). Following resection, a void is left within the breast. This cavity will not have the exact linear dimensions as the lumpectomy specimen due to the deformation of breast tissues under gravity, but may be held open using retractors to maintain a similar cavity volume as the lumpectomy specimen. The surface area of the cavity will be similar to that of the lumpectomy. Approximating the average cavity surface area to be an ellipsoid with the dimensions of average lumpectomies, we find that the surface area of the average surgical cavity is approximately 105.1 cm^2^. The device shall be capable of imaging tissues with these dimensions and geometries.

##### User and patient safety

In general, the device should be wipeable with intermediate-level disinfecting wipes to keep the device’s surface clean. During surgeries, the device is covered with a custom sterilized drape in order to be compatible with a sterile OR environment. The drape is sterilized by the Central Processing Department at University Health Network; Toronto, Canada (UHN) prior to its use in the OR (approved by UHN’s medical device reprocessing committee). The drape covers the entire device, including the imaging detector and illumination sources. It was learned during the PRODIGI trial [12] that off-the-shelf drapes, made of plastic material that added blur and other optical distortions to the image, are not suitable for high-quality imaging. The most suitable drape design would include a form-fitted portion secured to the device’s optical components, with sufficient rigidity and clarity through which imaging is minimally obstructed. With the surgical drape attached, a slight decrease in the quantity of light transmitted through this optical window to the imaging field and detector is expected. After each use, the surgical drape is discarded and the device surface is wiped using intermediate-level disinfecting wipes, such as CaviWipes (Metrex, Orange, CA, USA). A newly sterilized drape is used for the next surgery.

#### Imaging

Imaging aims to differentiate breast tissues at the surgical margins, both within the surgical cavity and on the excised specimen. The tissues of interest are healthy breast adipose tissues, connective tissues (predominantly fibrous tissue that may or may not include benign breast epithelial structures), and carcinoma remaining at the surgical margins post-resection. The residual carcinoma is identified by red FL, which is due to the presence of excess PpIX after administration of 5-ALA. These malignant tissue foci may have sub-millimetre dimensions. They may also have sub-millimetre thicknesses if present at the surgical margin. In the case of ductal carcinoma in situ (DCIS), clinically relevant positive margins include DCIS within 2 mm of the surface [40]. PpIX fluoresces red when illuminated with 405 nm light and imaged with a detector sensitive to PpIX’s emission wavelengths.

##### Illumination

Since PpIX-positive surgical margins may include low levels of FL due to the potentially sub-millimetre dimensions of residual disease, maximizing PpIX contrast against healthy tissues is required. Maximizing the overlap between the PpIX excitation spectrum and the light source’s emission spectrum will optimally excite PpIX. However, such a broad light source may decrease the ability to differentiate other tissues, e.g. connective or adipose breast tissues, and carcinoma. Therefore, constraining the light source’s emission to below $\sim$450 nm provides ample spectral availability for imaging healthy breast tissues without excitation light intrusion. The light source must, as a result, be easily filtered from the wavelengths of interest for imaging.

LEDs are the most suitable light source. They are compact, have large diffusion angles, and often have a modest full width at half maximum (FWHM), increasing the spectral overlap between the light source emission and PpIX excitation compared to a laser with single-wavelength emission. The LEDs selected must have peak wavelength $405\pm {10}\,$nm, with FWHM such that the maximum emission wavelength is <450 nm.

White LEDs are also required to provide the users with anatomical context during imaging. Switching between WL and FL imaging may help users colocalize suspicious regions discovered under FL imaging with the breast tissue as visualized under WL. The colour temperature of these LEDs must be cool, i.e. approximately $4000-{6000}\,{\text {K}}$ to avoid falsely saturating the dominant warm hues of breast tissues under WL and to better resolve details due to colour differences.

##### Optical filtering

For FL imaging, an optical filter is required to remove the excitation light from the image and transmit only the wavelengths of interest to the camera. The filter should be placed distally from the camera such that any light detected by the image sensor must first pass through the imaging filter.

**Fig. 1:**
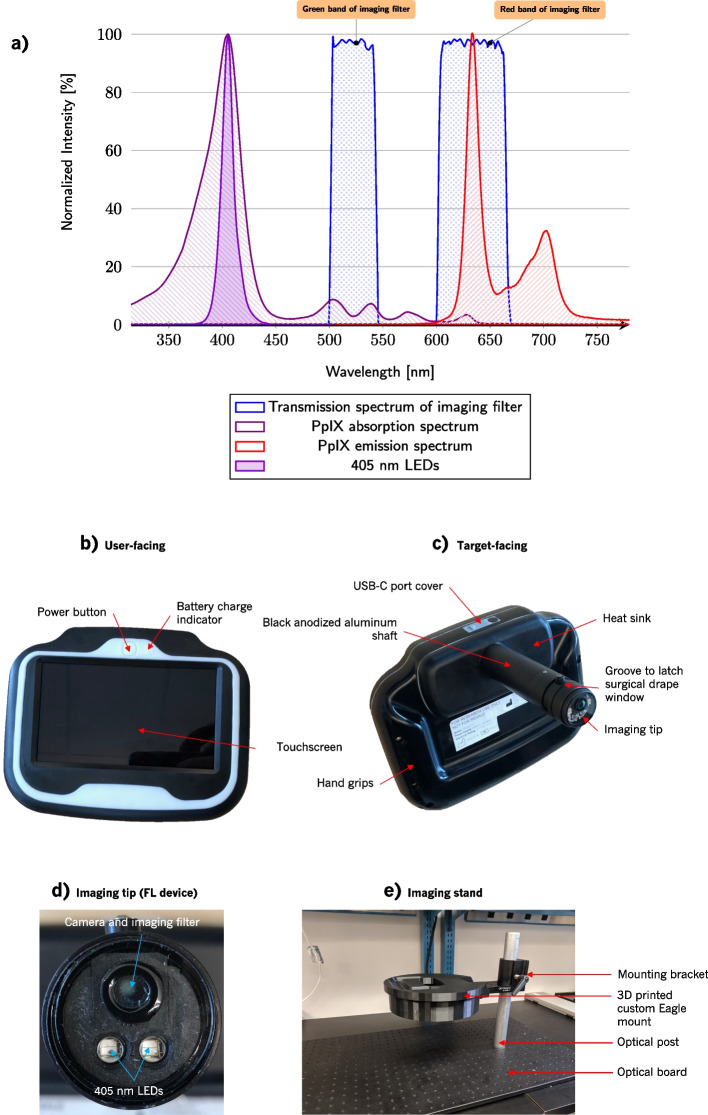
Eagle imaging device. **a** Spectral diagram of FL optical components and PpIX excitation and emission spectra. The primary PpIX excitation peak is targeted with 405 nm LEDs. The imaging filter transmits green (500 to 550 nm) and red (600 to 660 nm) wavelengths to the camera. **b** Front (user-facing) and **c** rear (target-facing) views of the Eagle FL prototype device. The WL device is physically identical aside from the removal of the emission filter at the imaging tip. The user-facing side features a built-in touchscreen display, which is used to capture and view images and videos. The optics are mounted on the target-facing side at the distal tip of an aluminum shaft, which is sized to reach into and image all surfaces of surgical cavities. **d** Imaging tip of the Eagle FL device showing relative positions of the LEDs and camera. The diameter of the imaging tip is 24 mm. **e** Eagle imaging stand used to mount the Eagle device for consistent, reproducible ex vivo imaging. The Eagle prototype, mounted in the 3D printed custom mount, sits with the optics pointed directly downward at the imaging sample. A disposable, sterile black imaging sheet (not pictured) is taped down to the optical board when imaging biological tissues to avoid contamination of the sample and optical board. The imaging distance can be adjusted by sliding the mounting bracket along the optical post and measuring the distance between the Eagle device’s tip and the sample. The post is graduated and keyed to prevent rotation of the mounting bracket

To image PpIX and breast tissues following 5-ALA administration and with 405 nm excitation LEDs, we require an optical emission filter with a stopband to block the LED emission and a passband to transmit PpIX emission. During the PRODIGI trial, we found that including a green passband helped to contrast PpIX against the surrounding healthy connective tissues [12]. Connective tissues have a broad emission spectrum, and without this green band, the red portions of connective tissue FL emission would be difficult to visually distinguish from PpIX [41]. Therefore, the filter used in the PRODIGI device (Fig. 1a) is suitable for this application. This filter adequately blocks 405 nm excitation light while providing PpIX-to-normal tissue colour contrast.

##### Camera module

The goal of intra-cavity FL imaging during BCS is to identify small regions of malignant tissue that remain after the lumpectomy specimen is excised. These regions may be as small as a few tumour cells across or about 100 $\upmu$m. Therefore, the camera’s resolution must be able to visualize features of 100 $\upmu$m in size or smaller at a suitable imaging distance. The ideal imaging distance depends on the field of view (FOV) and illumination intensity. The distance should be sufficiently close to illuminate the imaging field with bright light but far enough away to capture a large portion of the imaging field within the FOV. Furthermore, imaging distances are likely to differ for intra-cavity and ex vivo imaging. Cavity imaging should be done from a small distance, e.g. 3 cm, due to the size constraints within the surgical cavity. Lumpectomies can be imaged from a further distance, e.g. 10 cm. It is desirable to capture the entire sectioned specimen in a single image; the FOV at 10 cm should be sized accordingly. The camera should be able to automatically focus on the imaging target at and between the cavity and lumpectomy specimen imaging distances.

The maximum FOV dimensions depend on the angle of view $\theta$ and distance from the imaging target *d*. *r* is the radius of the FOV circle in the imaging plane related to $\theta$ and *d* by Eq. 1:

1 $$\begin{aligned} \tan \left( {\frac{\theta }{2}}\right) =\frac{r}{d}. \end{aligned}$$

Assuming a 4:3 pixel aspect ratio, FOV dimensions *a* and *b* can be calculated as $a=4x$ and $b=3x$ where the value *x* is determined by Eq. 2:

2 $$\begin{aligned} r = \sqrt{\left( {\frac{a}{2}}\right) ^2+\left( {\frac{b}{2}}\right) ^2} = \sqrt{\left( {2x}\right) ^2+\left( {1.5x}\right) ^2} = 2.5 x. \end{aligned}$$

So, the maximum FOV dimensions are defined by Eq. 3:

3 $$\begin{aligned} a = 1.6d\tan \left( {\frac{\theta }{2}}\right) \qquad \text {and}\qquad b = 1.2d\tan \left( {\frac{\theta }{2}}\right) . \end{aligned}$$

With specimen length and width expected to be smaller than ${8.9}\,\text {cm}\times {9.9}\,\text {cm}$ in most cases (mean + standard deviation of two largest lumpectomy specimen dimensions), we can assume *b* needs to be greater than approximately 8.9 cm. At this dimension, $a={11.9}\,\text {cm}$. At a 10 cm specimen imaging distance, this requires $\theta >73.2^{\circ }$.

To match the 1 min cavity scanning requirement (10 s per cavity surface) and considering the 105.1 cm^2^ surface area of an average cavity, a scanning rate of at least $\frac{17.6\textrm{cm}^{2}}{10\,\text {s}}$ is required. With $\theta =73.2^{\circ }$, the FOV when imaging the cavity from approximately 3 cm is ${3.6}\,\text {cm}\times {2.7}\,\text {cm}$, which has area 9.7 cm^2^. With this FOV, significantly less than 10 s will be required to scan each cavity surface ($\sim$17.6 cm^2^). Therefore, these FOV dimensions and imaging distances seem reasonable.

For consistent imaging, and given that lighting conditions and imaging targets are similar across imaging sessions, image processing variables such as white balance, exposure, and *RGB* channel gains should be predefined and static across imaging sessions to optimize detection of PpIX. These parameters should be consistent during still imaging and video capture. Fixing the exposure time *t* creates a maximum frame rate limit $f_{\text {max}}$ in the form $f_{\text {max}}=\frac{1}{t}$. For smooth (i.e., not choppy) video, we require the average frame rate $\overline{f}$ to be $\overline{f} > 24$ frames per second.

### Clinical testing

#### Clinical trial patients

PMCC (Research Ethics Board (REB)# 10-0633-CE) and Hospital; Toronto, Canada (MSH) (REB# 13-0155E) approved the clinical protocol for this study (ClinicalTrials.gov Identifier: NCT01837225). All patients provided written informed consent before study participation. Patients were screened for eligibility by research staff during their pre-operative clinic visits. The inclusion and exclusion criteria are listed in Table 1. Thirty-two patients consented to participate in the study, of which eleven were withdrawn before data collection due to treatment plan changes and COVID-19- and administrative-related reasons. Four additional patients were withdrawn following incomplete or poor-quality data collection due to time constraints within the clinic. Participants underwent standard resection of the primary lumpectomy specimen and, if necessary, margin resections, followed by FL imaging of the surgical cavity and specimen by a researcher. Biopsies from the surgical cavity were collected by the surgeon and biopsies from the surgical specimen were collected by the PA.

**Table 1:** Inclusion and exclusion criteria for the clinical trial

Inclusion Criteria	
1.	$\ge 18$ years of age
2.	female
3.	diagnosed with primary invasive breast cancer or in situ ductal carcinoma
4.	palpable carcinoma
5.	consented for standard surgery for primary breast cancer
6.	planned to undergo BCS (lumpectomy or partial mastectomy)
7.	consented to banking of core biopsies with UHN tissue bank
Exclusion Criteria	
1.	preoperative therapy (endocrine therapy, chemotherapy, radiotherapy)
2.	currently on neoadjuvant therapy to treat another cancer
3.	undergoing mastectomy
4.	diagnosis of lobular carcinoma based on preoperative biopsy
5.	prior history of photosensitivity, liver disease, or recurrent disease
6.	history of renal and/or liver impairment
7.	pregnancy
8.	inability to consent

#### Administration of 5-ALA

5-ALA HCl (generously provided by photonamic GmbH and Co. KG, Pinneberg, Germany) was administered at $20\frac{\text {mg}}{\textrm{kg}}$ BW dosage 2-4 h before surgery. The same administration and adverse event documenting methods reported in the PRODIGI trial [12] were followed.

#### Intraoperative fluorescence imaging of the surgical cavity and excised specimen margins

The ability of the Eagle device to differentiate breast tissues in the surgical cavity immediately following lumpectomy or partial mastectomy was investigated among $n = 17$ patients. WL and FL imaging of the cavity was performed after resection of the primary lumpectomy specimen, including both before standard of care margin assessment (e.g. specimen radiology) and after resection of any additional tissue (e.g. revised margins). Both videos and still images were captured of the surgical cavity in the OR. If areas of PpIX red FL were observed on an anatomical surface of the cavity (posterior, lateral, inferior, medial, superior, anterior), at least one FL image was taken of that surface. WL and FL images of each of the six anatomical surfaces of the excised specimen were collected in the operating room. If areas of PpIX red FL were observed on the excised primary specimen, they were tagged using a suture. In all cases, a control biopsy from the surgical cavity (in an area of non-PpIX red FL) was collected. The biopsy was placed on a designated sterilized black polyoxymethylene imaging sheet (dimensions: $12.5\times {15}\,\text {cm}$) and a FL image was captured of the side of the biopsy for cataloguing. Before imaging, surgical cavity dimensions (incision length, opening length and width, and cavity depth) were measured using a sterile ruler.

#### Ex vivo fluorescence imaging of the sectioned lumpectomy specimen

Following BCS, surgical specimens were sectioned and imaged in the pathology suite. Margins were painted by the PA using a standardized margin inking schema, specimens were serially sliced grossly (“bread-loafed”), and the slice containing the largest clinically discernible cross-section of the tumour was laid open. The specimen was placed on a black imaging sheet ($25\times {30}\,\text {cm}$) on the optical board of an imaging stand (Fig. 1e). The specimen was centred in the camera’s FOV at the middle of the two slices. The distance between the Eagle device’s camera and the surface of the specimen was adjusted to be 10 cm using the mounting bracket of the imaging stand. If the tumour slice did not fit within the FOV of the camera when laid open at this imaging distance, the camera was centred on one of the tumour slices. WL images were collected under dark room lighting conditions (<0.01 lx) with the white LEDs turned on. FL images were then collected, ensuring each WL image had a spatially colocalized FL image. FL images were acquired under the same dark room light conditions, consistent across imaging sessions. WL and FL image acquisition took <1 s and 1-2 s, respectively per image. Biopsies were collected following imaging. One 2 mm punch biopsy was collected from within the PA-defined tumour border (if the tumour measured $\ge {1.7}\,\text {cm}$ in diameter during gross assessment by the PA) and up to three 4 mm biopsies were collected from outside the tumour border. For biopsies collected within the tumour border, the PA was blinded to FL images. For biopsies collected outside the tumour border, research staff guided biopsy selection based on FL appearance to maximize the diversity of FL colours represented in the collected biopsies. The imaged surface of each biopsy was inked. Specimens were placed in formalin for fixation within 1 h of surgical excision as per clinical practice.

#### Histopathologic analysis

Research tissue biopsies were collected by surgeons from the surgical cavity and by study research staff from ex vivo specimens for gold standard histological evaluation by the study pathologist blinded to the imaging results. Each biopsy core was fixed in formalin and individually placed into a standard histopathology cassette, labelled, and submitted for processing, sectioning, and hematoxylin and eosin (H&E) staining. All stained sections were reviewed by the blinded study pathologist to catalogue the tissue types, to determine the presence of invasive and/or in situ carcinoma, and to evaluate relative tissue proportions in each biopsy for correlation with FL imaging.

#### Image analysis

A major focus of this study was to evaluate the strengths and limitations of the Eagle device to differentiate breast tissue types, including breast tumours, following administration of $20\frac{\text {mg}}{\textrm{kg}}$ BW 5-ALA. $\Delta E_{2000}$ (simplified to $\Delta E$) was calculated using Matlab (MathWorks, Natick, MA, USA) to quantify the colour contrast between tissues. This quantity, developed by the International Commission on Illumination (CIE), can be used to quantify the perceptible difference between two colours, where $\Delta E\approx 1$ is defined as the minimum perceptible colour change by humans [42]. Generally, for this study, $\Delta E\le 10$ represents colours that are substantially similar and have low contrast, $10<\Delta E< 50$ represents colours that can be visually differentiated with reasonable contrast, and $\Delta E\ge 50$ implies colours that are different with high contrast. Therefore, $\Delta E$ should be low for colour-matching purposes, and for adequate colour contrast, $\Delta E$ should be high. In addition to measuring the contrast between tissue types, $\Delta E$ was also used to measure colour changes due to image artifacts or in response to new imaging conditions. $\Delta E$ was calculated using two colours as inputs [43, 44]. To calculate the contrast between two regions of interest (ROIs), the average colours of each ROI were calculated, and the $\Delta E$ between these two colours was subsequently calculated.

#### Phantoms to evaluate imaging device design

##### Surgical drape

To investigate the impact of the drape on imaging, a pork tissue phantom was constructed. Pork tissue was chosen due to its similar appearance to human breast adipose tissues when imaged with the Eagle device. Six wells were cut into this phantom in a circular pattern using a 4 mm punch biopsy tool. Each well was filled with 630 nm quantum dots (Sigma-Aldrich) diluted in optimal cutting temperature gel to prevent the fluorophore from diffusing into the pork tissue. The quantum dot concentrations used were 10, 20, 39, 78, 156, and ${312\frac{\upmu \text {g}}{\textrm{mL}}}$.

##### Illumination uniformity

The uniformity of the Eagle device was determined by measuring the 405 nm optical power density from 3 and 10 cm distances with an optical power meter (Pronto-Si, Gentec-EO, Quebec City, QC, Canada) at nine locations within the FOV ($3\times 3$ grid pattern including the corners and centre of the FOV). To increase the resolution of these measurements and incorporate effects of the camera and imaging filter on uniformity, if any, the uniformity was also measured by imaging a wide dish ($17\times {17}\,\text {cm}$) filled to a 1 cm depth with 5 $\upmu$ m PpIX (Sigma-Aldrich) in dimethyl sulfoxide (DMSO) (Sigma-Aldrich) from 3 and 10 cm. Then, the pixel-wise intensity map of each image was used to calculate how the intensity falls off toward the edges of the FOV and plotted as contours. The Eagle FL device was mounted in its stand during power measurements and imaging.

## Results

### Imaging device design

The Eagle imaging device was manufactured in two different configurations to simplify the optical design: one version for FL and a separate version for WL imaging. Future iterations will include both imaging modes in a single device. The FL device has 405 nm LEDs and an imaging filter, while the WL device has white LEDs and no imaging filter. The devices are otherwise identical.

#### Form factor and user interface

The Eagle device was designed to prioritize intra-cavity imaging capabilities while retaining the ability to image ex vivo specimens. Designed as a fully wireless, handheld device shown in Fig. 1b and c, the Eagle device (bounding box dimensions $17\times 13\times {12}\,\text {cm}$, mass 557 g) consists of a black anodized aluminum shaft (diameter 24 mm, length 91 mm) connected to a body containing the battery, electronics, and touchscreen interface. The optical components are mounted on the distal end of the aluminum shaft at the “imaging tip” labelled in Fig. 1c. The user interface is based around a built-in colour organic LED (OLED) touchscreen, which is used to control the camera and LEDs, capture images and videos, and review collected data. The OLED screen enables the display of deep black levels and can achieve a high contrast ratio in low ambient light conditions, as required during FL imaging. The brightness of the display is fixed. The Eagle device is powered by a rechargeable Li-ion battery.

#### Imaging

The Eagle device’s principle of operation is similar to that of the PRODIGI prototype [12]. Similar optical components are inbuilt to enable visualization of healthy and malignant tissues within the breast. The optical components in the Eagle FL device include a camera module, 405 nm LEDs, and an imaging filter.

##### Illumination

To excite PpIX, the optimal excitation wavelength is 405 nm as shown in Fig. 1a. The Eagle FL device has two $405\pm {15}$ nm bandwidth at FWHM LEDs with spectrum shown in Fig. 1a. This excitation wavelength also facilitates the excitation of connective tissues. The Eagle WL device has two 5000 K white LEDs.

##### Optical filtering

The imaging filter, distal to the camera module, has the same transmission spectrum as the filter built into PRODIGI (green: 500-545 nm and red: 600-660 nm, see Fig. 1a). This imaging filter was selected to block the excitation light (optical density (OD) $\ge 6$ between 300 and 492 nm) while transmitting red and green wavelengths to the image sensor. The red bandpass region is primarily for visualizing the red FL emitted from the cancerous cells and secondarily contributes to the visualization of breast adipose tissues. The green bandpass region is for visualizing FL emission within the green pass-band from connective tissue components such as collagen.

##### Camera module

Both versions of the Eagle device (FL and WL) include the same camera module—an 8 MP complementary metal oxide semiconductor (CMOS) image sensor (OmniVision, Santa Clara, CA, USA) and a lens assembly facilitating autofocus between $d=2$ and 15 cm. The camera module has an angle of view of $\theta =85^{\circ }$. The white balance of the camera is static and consistent between images. When capturing FL videos, the frame rate is dynamic based on the fixed exposure and ranges, on average, from 24 to 30 Hz. The spectral range of the image sensor is 400 to 1000 nm. The camera’s resolution at 3 cm is ${5.6}{\frac{\text {lp}}{\textrm{mm}}} \approx {89}{\upmu }$m.

##### Configuration of optical components

The imaging tip of the Eagle FL device is shown in Fig. 1d. The imaging filter is positioned distally from the camera such that all light transmitted to the camera passes through the filter. Securing the filter is a 1.1 mm-thick circular fused silica window with a 6 mm-diameter hole cut for filter insertion. The window protects the optical components and provides a highly transmissible, wipeable surface. There is no filter on the WL device, so the window shields the camera and the white LEDs. On both configurations of the Eagle device, the camera is isolated by a baffle to prevent internal reflections from being captured by the image sensor. The image sensor is 6 mm proximal to the optical window. The two 405 nm LEDs are arranged toward the bottom edge of the camera sensor. The uniformity of this positioning is poor, specifically at the top of the FOV. Additionally, LED power output, beam angle, and proximity to the camera resulted in violet excitation light bleeding into the bottom of the FOV. To mitigate this and improve the uniformity, the FOV width and height were both cropped to $68\%$ of their maximum possible dimensions.

##### Surgical drape

Custom disposable drapes were designed to fully enclose the Eagle device for use within the surgical field. The drapes each include an optically transparent polycarbonate window attachment (thickness: 1.8 mm, average light $\%T$ at $0^{\circ }$ angle of incidence (AOI): $92\%$, average light $\%T$ at $45^{\circ }$ AOI: $88\%$) to preserve the image quality and minimize optical distortions observed while imaging with PRODIGI in the OR. The optical window clips onto the Eagle device at the groove labelled in Fig. 1c. Drapes were sterilized with ethylene oxide gas by the Medical Device Reprocessing Department at UHN before use in the OR.

##### Imaging stand

An imaging stand (Fig. 1e) with height adjustment was developed to ensure consistency across imaging sessions. The stand allows the Eagle device to be secured in the 3D printed mount with the camera and LEDs pointed down at the imaging sample placed on the optical board. The height of the imaging device on the stand can be adjusted by sliding the mounting bracket along the optical post and locking the bracket into place. The keyed, millimetre-graduated post prevents rotation of the mounting bracket and facilitates simple and consistent imaging distance adjustments.

### Device usability

#### Eagle usability in the clinic

The Eagle prototype device generally performed well in the clinic. The device’s size and shape facilitated the ability to image every surface of surgical cavities. However, the device’s mandatory two-handed use required a second user to manipulate the cavity to expose each surface during imaging. Due to the rigid design of the device, imaging the lateral surfaces of surgical cavities required aiming the device’s screen away from the user. A second user was required to read the screen from the opposite side of the patient, or a combination of cavity and user contortions was required for the imager to view the screen while imaging the lateral cavity margin.

A single cavity scan took approximately 2-2.5 min. Image capture time took 1-2 s, often resulting in blurry images due to motion during image capture. In addition, the display screen was covered by the drape, and a preview of the captured image was not immediately shown to the user. Without an immediate image preview, it was difficult for the user to determine if the captured image was blurry and if the image had to be recaptured.

Draping the device was a time-consuming procedure due to the difficulty of drape installation. Ensuring the plastic drape material was installed to fully cover the device without compromising sterility required careful unfolding of the drape and coordination between three users (two scrubbed, one non-sterile). The drape was also observed to interfere with and decrease the sensitivity of the touchscreen display. Finally, the drape produced reflections in lit rooms, which made viewing the screen difficult.

#### Surgical cavity dimensions

The dimensions of BCS surgical cavities ($n=17$) were measured to understand the suitability of the Eagle device’s form factor for imaging within these confined spaces.

Surgical cavities had mean incision length ${5.63}\,\text {cm}$ (range: 3.5-9 cm, standard deviation: 1.62 cm). After the cavities were created, the openings were approximately oval with mean long axis length ${4.83}\,\text {cm}$ (range: 3-8 cm, standard deviation: 1.14 cm) and short axis length ${4.48}\,\text {cm}$ (range: 2-9 cm, standard deviation: 1.78 cm). On average, cavity depths were ${3.33}\,\text {cm}$ (range: 1.5-7 cm, standard deviation: 1.39 cm).

### Imaging findings

The Eagle device was introduced into a clinical trial to gain an understanding of the Eagle device’s functionality within a BCS clinical environment rather than to measure the explicit performance of the device. The trial was designed to highlight the most critical areas for improvement by exposing the device to the intended users, workflows, patients, and imaging targets. In the clinic, we discovered strengths of the Eagle device and areas for future development. Specifically, we were able to use the Eagle device to differentiate healthy (adipose, connective) and cancerous tissue following 5-ALA administration. We also discovered several imaging artifacts due to the device design, surgical drape, and factors specific to ORs.

#### Tissue differentiation

**Fig. 2:**
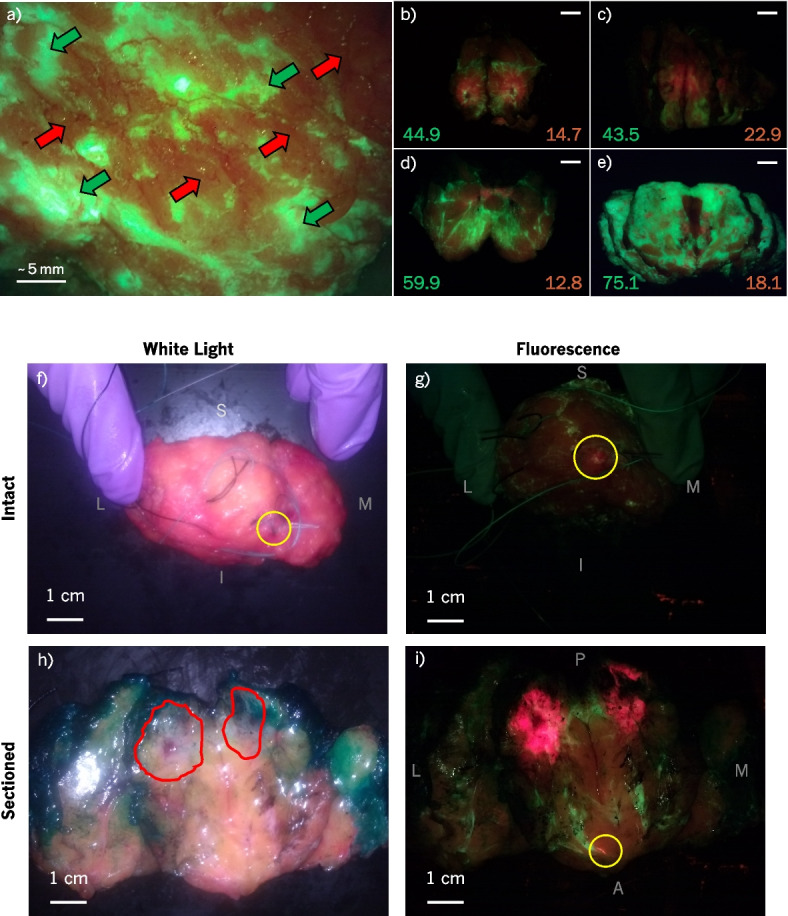
Breast tissues imaged intraoperatively with the Eagle FL device. **a** Breast surgical cavity imaged intraoperatively with the Eagle device from a distance of approximately 3 cm. Based on past experience correlating breast FL images with histopathology and biopsy samples collected in this study, this FL image shows connective tissue in green (green southwest-facing arrows) and adipose tissue in orange-brown (red northeast-facing arrows). **b**-**e** Sectioned lumpectomy specimens imaged with the Eagle FL device from 10 cm. $\Delta E$ was calculated between a red ROI and suspected connective and adipose regions in each sectioned specimen. $\Delta E$ values are listed in the corners of b-e between the PpIX and connective in the bottom left and PpIX and adipose in the bottom right. IDC was confirmed in biopsies taken within the tumour borders of (**b**)-(**d**); **e** was diagnosed as DCIS so no biopsy was taken. Scale bars in (**b**)-(**e**) are 1 cm. **f**-**i** WL and FL images of a lumpectomy specimen from a BCS patient with a positive margin detected during intraoperative imaging. **f** and **g** show the anterior surface of an intact specimen held in place with a gloved hand, while **h** and **i** show the specimen sectioned with the tumour cross-section laid out. Anatomical surfaces are labelled as follows: superior (S), inferior (I), medial (M), lateral (L), posterior (P), anterior (A). The primary tumour mass, within the red border drawn in (**h**), fluoresced bright red as shown in (**i**) in contrast to the green and orange-brown healthy tissues. The areas circled in yellow in **f**, **g**, and **i** indicate a DCIS positive margin that was missed by the standard of care but appeared red under FL using the Eagle imaging device. Surgical pathology confirmed the presence of DCIS <1 mm below the surface, explaining the lack of corresponding FL in the surgical cavity

Using the Eagle device, we were able to differentiate between adipose and connective breast tissues based on FL appearance and confirmed by correlative histopathology. Connective tissues appeared bright green, while adipose tissues appeared orange-brown, as shown in Fig. 2a. Past experience identifying these tissue types based on FL that was gained in the PRODIGI trial [12] guided identification of the tissue types when imaging with the Eagle device in the present clinical trial. A total of 8 green fluorescent biopsies from 6 patients and 14 orange/brown biopsies from 11 patients were collected from the sectioned specimens and surgical cavities. In $n_{\text {green}}=8$ biopsies suspected to be connective tissues based on FL imaging, seven had connective tissue proportion $\ge \!50\%$ in the biopsy section analyzed. The lone false positive was due to the presence of Patent Blue V dye in the breast tissue, causing adipose tissue to appear dark green (i.e., like neither connective nor adipose tissue but more similar in appearance to connective than adipose). $100\%$ of $n_{\text {orange/brown}}=14$ biopsies suspected to predominantly contain adipose tissues based on FL imaging had adipose tissue proportion $\ge \!50\%$.

In addition to differentiating healthy breast tissues, we were able to visualize breast tumours in sectioned lumpectomies using the Eagle device. Figure 2b-e show four examples of sectioned lumpectomies with connective tissues (green), adipose tissues (orange-brown), and PpIX-containing tumours (red). $\Delta E$ was calculated between the red and suspected connective regions (bottom left of Fig. 2b-e), as well as between the red and suspected adipose regions (bottom right of Fig. 2b-e). On average, the colour difference between red tumour and connective tissues among these four examples is $\overline{\Delta E} = 55.9\pm 14.8$ and $\overline{\Delta E} = 17.1\pm 4.4$ (mean ± standard deviation, $n=4$) between tumour and adipose tissues. Specimens b-d all contained invasive ductal carcinoma (IDC) in biopsies taken from within the tumour region defined by the PA. Specimen e was diagnosed as DCIS; therefore, biopsies were not obtained. However, red FL from PpIX is visible, and surgical pathology confirmed the presence of DCIS within the lumpectomy specimen.

Figure 2i shows another example of bright red FL emanating from the tumour region, demarcated in Fig. 2h. In addition to the IDC found within the tumour border in Fig. 2i, we also identified DCIS at the surgical margin, which was not discovered by the intraoperative standard of care. This region was identified on the anterior margin of the intact specimen immediately post-resection, shown in Fig. 2g. After the lumpectomy specimen was sectioned, we were able to visualize the cross-section of this suspicious region as shown in Fig. 2i. According to the surgical pathology report, the DCIS was $<1\,\text {mm}$ below the surface of the lumpectomy specimen, which is a positive margin according to SSO-ASTRO guidelines [40]. We were able to visualize this positive margin using the Eagle device immediately following resection.

We also discovered several areas for Eagle device improvement through rigorous clinical testing. This trial allowed us to gain exposure to the clinical environment and adapt the Eagle imaging system and workflows to the characteristics of this setting. For example, we discovered imaging artifacts produced by the surgical drape while imaging surgical cavities.

#### Surgical drape

The surgical drape was designed to improve over the PRODIGI drape, which was a long sheath secured to the PRODIGI device using sterilized neodymium magnets. The drape material through which images were captured reduced image quality by adding blur and distortion artifacts. The Eagle drape was, therefore, designed to incorporate a rigid optical window secured to the tip of the device for consistent image quality.

**Fig. 3:**
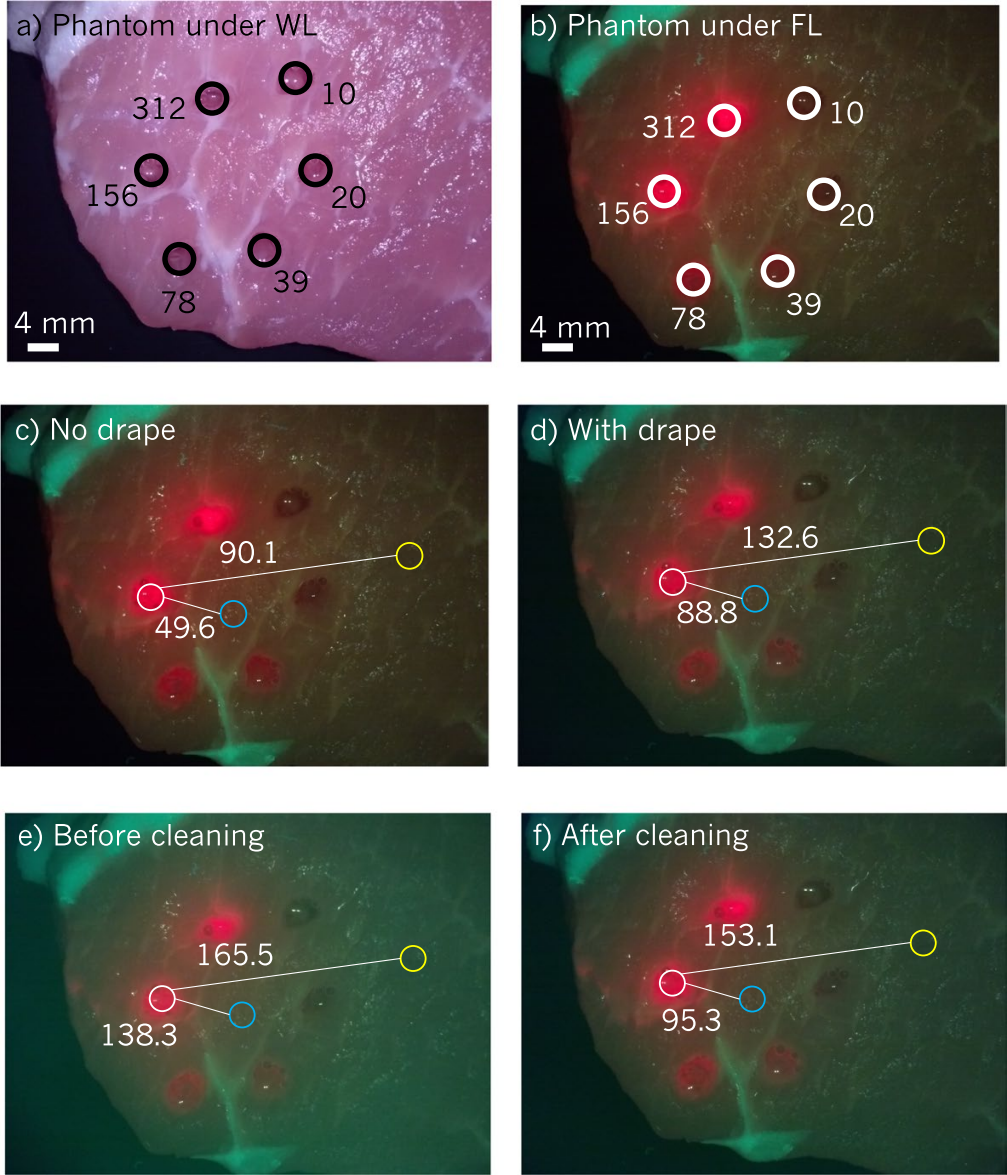
Pork tissue phantom imaged with the Eagle device to evaluate image quality with the surgical drape. **a**-**b** Pork tissue phantom constructed to investigate the impact of the surgical drape on imaging. Wells were cut into the pork tissue with a 4 mm punch biopsy tool and filled with 630 nm quantum dots in the concentrations shown $\left[ \frac{\upmu \text {g}}{\textrm{mL}}\right]$. **c**-**d** Pork tissue phantom imaged **c** without and **d** with the surgical drape installed. The values adjacent to white lines represent the $\Delta E$ between the average colours within the circles connected by the white lines. Contrast between the quantum dots and pork tissue increased with the surgical drape installed due to a green tint produced by the drape’s optical window. **e**-**f** Pork tissue phantom imaged **e** before and **f** after cleaning the optical window with lens cleaner. Contrast between the quantum dots and pork tissue decreased after cleaning but remained sufficient to distinguish quantum dots from healthy tissue. Image quality, however, increased after cleaning due to more accurate colour representation and better clarity

The pork tissue phantom described in Phantoms to evaluate imaging device design section and shown in Fig. 3a and b was imaged from approximately 6 cm with and without the drape installed on the Eagle device, as shown in Fig. 3c and d. With the drape installed, a green tint is evident when imaging with the surgical drape. This tint is due to green FL from the optical window material and the epoxy securing the optical window to the drape cap reflecting into the camera sensor. The green tint provided by the drape’s window serves to *increase* the contrast between the red quantum dots and pork tissue as shown in Fig. 3c and d. The contrast between the ${156}\frac{\upmu \text {g}}{\textrm{mL}}$ quantum dot well and two areas on the pork tissue were calculated: one region central to the FOV, and one near the FOV edge where effects of illumination non-uniformity are increasingly apparent. $\Delta E$ in both cases increased when adding the drape (centre from 49.6 to 88.8, $79.0\%$ increase; right side from 90.1 to 132.6, $47.2\%$ increase).

The cleanliness of the drape’s window was also found to influence the green tint, as shown in Fig. 3e and f. Windows covered with fingerprints, smudges, or other debris were observed to increase the green tint. Following cleaning the window with a lens cleaning solution, the image quality increased without sacrificing sensitivity to red FL.

**Fig. 4:**
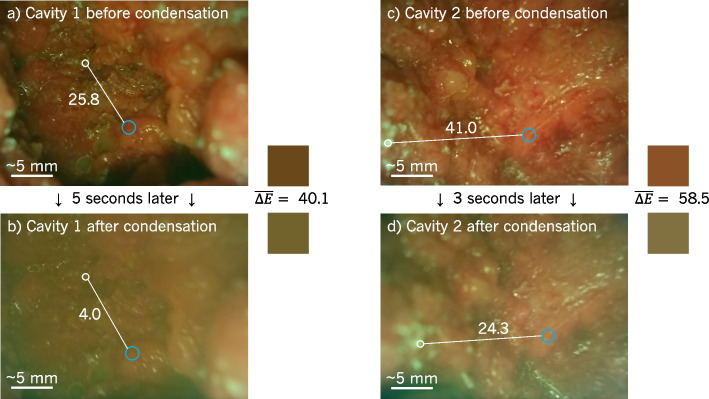
Haze produced while imaging within the surgical cavity. Two surgical cavities shown. Images of each cavity were captured before and after condensation buildup on the optical window of the drape (3-5 s later). Change in average colour (coloured squares beside each image) of each cavity had $\overline{\Delta E} > 40$ following condensation. Image quality also suffered following condensation buildup: details obscured and colours of all tissues converged to brown-green. In each image, $\Delta E$ between an adipose and connective tissue region were calculated; the addition of the haze reduced the contrast between these tissue types in both cavities

An unexpected consequence of imaging within the surgical cavity was the buildup of condensation on the optical window of the surgical drape, causing images to appear hazy, as shown in Fig. 4. The examples from two cavities demonstrate the dramatic change in appearance within seconds of imaging due to condensation. After 5 s, the average colour of cavity 1—shown in the small square to the right of Fig. 4a—changed with $\overline{\Delta E}=40.1$. Similarly, the average colour of cavity 2 changed by $\overline{\Delta E}=58.5$ after 3 s of imaging the cavity. This is a significant colour change solely due to condensation. Furthermore, the condensation reduced the colour contrast between adipose and connective tissues as evidenced by Fig. 4. In cavity 1, $\Delta E$ between adipose connective ROIs decreased from 25.8 to 4.0, an 84.5% decrease in contrast. In cavity 2, the decrease in contrast was by 40.7% ($\Delta E$ decrease from 41.0 to 24.3). Removing the device from the surgical cavity for 2 to 5 s before reinserting provided sufficient time for the condensation to dissipate. The condensation produced the most haze during the first $\sim$2 min of imaging, after which it became barely noticeable.

#### Illumination uniformity and imaging field of view

**Fig. 5:**
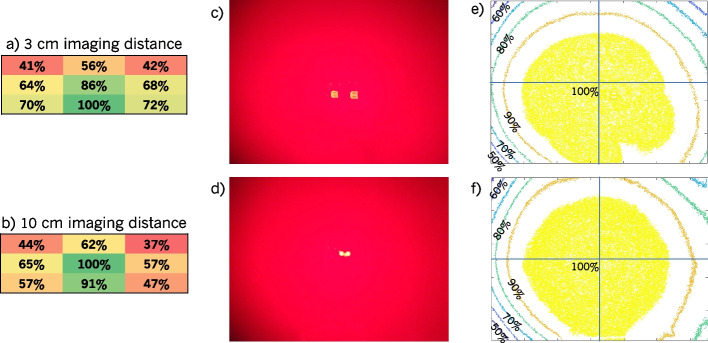
Eagle device FL illumination uniformity. **a**-**b** Relative optical power measured at nine evenly-spaced $1\times 1\,\text {cm}$ locations within the FOV from 3 and 10 cm imaging distances. Percentages are relative to the maximum optical powers measured: ${71.1}\frac{\text {mW}}{\textrm{cm}^{2}}$ from 3 cm and ${9.5}\frac{\text {mW}}{\textrm{cm}^{2}}$ from 10 cm. **c**-**f** Eagle FL device illumination uniformity measured by imaging a wide dish of liquid PpIX from 3 cm (**c** and **e**) and 10 cm (**d** and **f**). Images (**c**) and (**d**) were captured of the PpIX dish. Reflections from the LEDs are visible near the centres of the FOV. Intensity contours were calculated from these images in steps of $10\%$ relative to the maximum intensity, shown in panels (**e**) and (**f**). Crosshairs denoting the centre of each FOV axis were overlaid to observe the offset of the illumination from the centre. The “notch” in the lower right corner of the $100\%$ contour in panel (**e**) is likely due to movement of the PpIX liquid during image capture

The uniformity of the Eagle FL device after cropping the FOV, determined by measuring the 405 nm optical power density from 3 and 10 cm distances with an optical power meter is shown in Fig. 5a and b. From a 3 cm distance, the maximum power was recorded along the bottom edge, which may be expected due to the relative positions of the LEDs and camera. In both cases, the regions of the highest power density were focussed near the bottom of the FOV rather than the top. The uniformity was also measured by imaging a dish filled PpIX from 3 and 10 cm shown in Fig. 5c and d respectively. The resulting uniformity contours are shown in Fig. 5e and f. The uniformity according to this data is improved compared to the power meter measurements shown in Fig. 5a and b. The improved uniformity may be due to additional light reflection throughout the liquid, causing greater excitation and, therefore, more PpIX FL emission at the edges than caused by direct illumination from the Eagle device. These contours confirm the tendency of the illumination toward the bottom of the FOV, especially at smaller imaging distances.

**Fig. 6:**
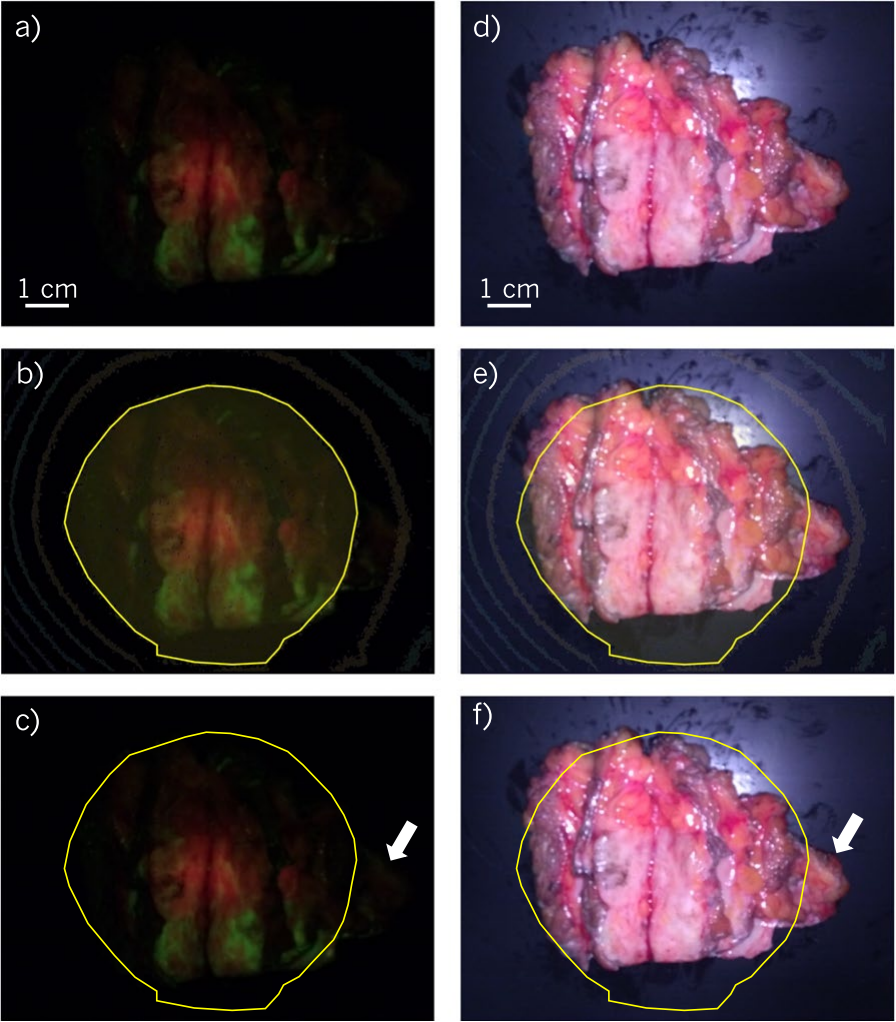
Uniformity as it impacts imaging ex vivo specimens from large imaging distances. Image **a** was captured of the sectioned specimen using the FL device and **d** using the WL device. In **b** and **e**, the uniformity contours from Fig. 5f were overlaid on (**a**) and (**d**) respectively. The outline of the maximum uniformity contour was traced by the yellow line. The contours were then removed from (**b**) and (**e**) to produce (**c**) and (**f**) respectively, leaving the traced maximum uniformity contour. A small piece of tissue outside of this contour, visible in (**f**) as indicated by the white arrow, is not obvious in **(c)**

The illumination uniformity affects the image quality, especially when imaging ex vivo specimens from large distances ($\sim$10 cm). The impact of uniformity is shown in Fig. 6, where a relatively large lumpectomy specimen was imaged from 10 cm. Figure 6a and d show the specimen imaged under FL and WL, respectively. In Fig. 6b and e, the uniformity contours from Fig. 5f were overlaid, and the $100\%$ contour was traced with the yellow line. The contours were removed in Fig. 6c and f, leaving only the $100\%$ contour trace. Outside of this region, the uniformity begins to fall off, resulting in the small region of tissue labelled by the white arrow in Fig. 6f to become nearly undetectable by FL in Fig. 6c.

The illumination uniformity informs decisions regarding the optimal imaging distance and FOV. The Eagle device has FOV cropping implemented to improve the uniformity. Each dimension of the FOV has been cropped to $68\%$ of its largest possible value resulting in only $46\%$ of the available area remaining in the FOV. This amount of cropping has shrunken the FOV such that imaging sectioned lumpectomies must be done from 10 cm away (to include the entire dimensions of most specimens in the FOV), thereby reducing the amount of light that excites the imaging target.

**Fig. 7:**
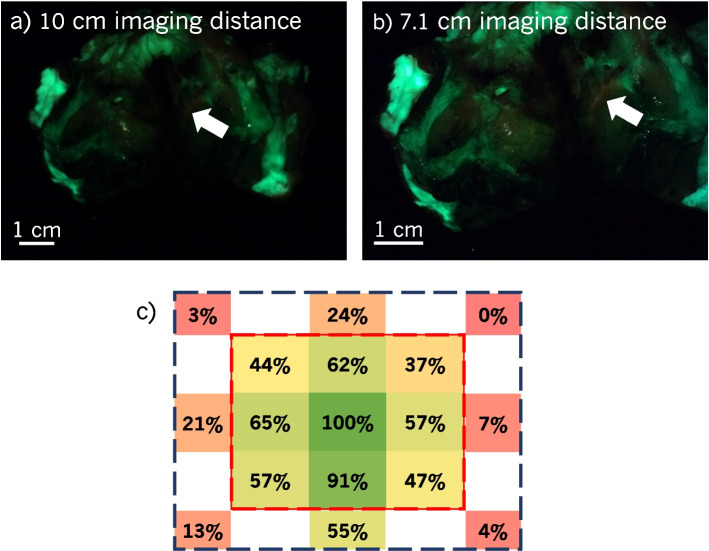
Impact of uniformity and FOV. **a**-**b** FL obscured due to the imaging distance and optical power. **a** was captured from 10 cm and **b** from 7.1 cm. The small region of PpIX at the tip of the white arrow was barely visible from 10 cm but more obvious from 7.1 cm. **c** Illumination uniformity from 10 cm. Within the cropped FOV (inner red rectangle), the uniformity is as shown in Fig. 5b. Outside of this region, and within the maximum potential FOV (outer blue rectangle), the uniformity suffers around the top and sides of the FOV due to the placement of the LED

The large imaging distance and decreased optical power can obscure essential features, as shown in Fig. 7a and b. From 10 cm, the PpIX at the white arrow in Fig. 7a is barely visible and may be missed if only imaged from this distance. From 7.1 cm, it is more distinguishable. With a larger FOV, the entire specimen can be imaged from a closer distance with a higher optical power.

To further illustrate the importance of improving the uniformity, the uniformity in the current FOV (red, inner rectangle in Fig. 7c) and the FOV before cropping (dark blue, outer rectangle in Fig. 7c) from a 10 cm imaging distance demonstrates the necessity of cropping with the configuration of the Eagle prototype’s imaging tip. In the larger FOV, the top corners receive $\le 3\%$ of the illumination compared to the centre. However, in areas of the FOV closest to the LEDs (i.e., along the bottom of the largest FOV), the relative power is greatly improved at $55\%$. Rearranging the LEDs so they surround the camera would be critical to improving the uniformity, usable FOV size, and optimizing the imaging distance.

#### Blood in the surgical cavity

**Fig. 8:**
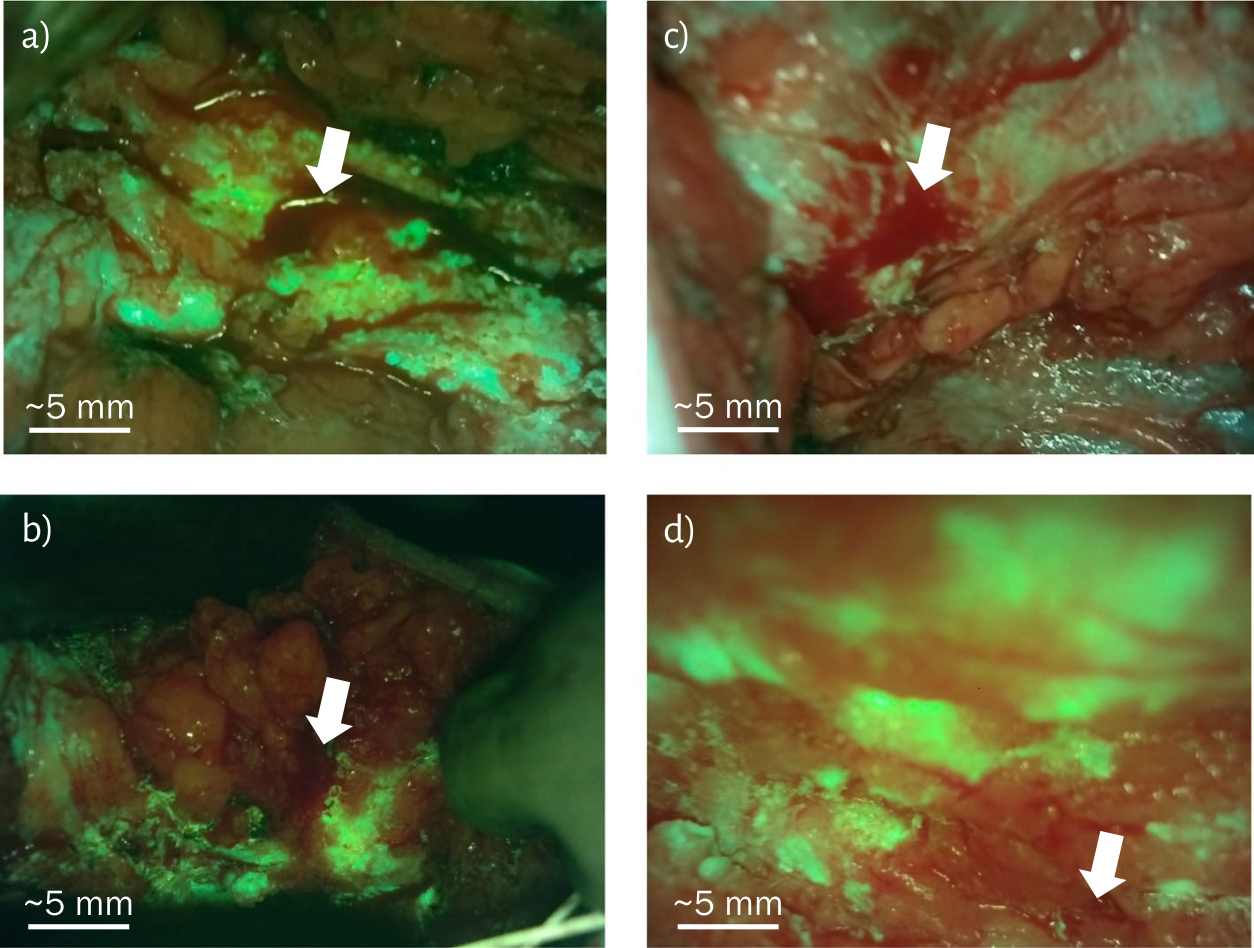
Blood in the surgical cavity as indicated by the white arrows. Blood may appear dark as in (**a**), (**b**) and (**d**), or brighter red as in (**c**), likely due to decreased imaging distance and increased optical power compared to (**a**), (**b**) and (**d**)

During BCS, blood may accumulate in the surgical cavity. Due to its strong absorption of 405 nm light, blood should be blotted or suctioned from the cavity before imaging to avoid obscuring FL from PpIX or breast tissues. Bleeding may continue during imaging, so the appearance of blood within the cavity and its impact on imaging should be considered. Blood normally appears dark red when imaged with the Eagle device in the surgical cavity, as shown in Fig. 8a, b, and d. In some cases, as in Fig. 8c, it can appear more luminous and, therefore, similar to PpIX. This luminous red light reflecting off the blood may originate from the 405 nm LEDs, which emit some light within the 600 to 660 nm range of the red band of the imaging filter.

## Discussion

### Suitability of the Eagle Device within the clinical setting

The Eagle prototype device generally performed well in the clinic. Successful intraoperative detection of the positive margin shown in Fig. 2f-i demonstrates the potential utility of real-time FL margin assessment during BCS. The device’s size and shape facilitated the ability to image all surfaces of surgical cavities. A device with a one-handed design can simplify the two-handed imaging procedure and make it possible for one user to accomplish. Single-handed use would also simplify imaging all surfaces of intact lumpectomies with a single user: rotating the lump with one hand and imaging with the other. Otherwise, the device’s cavity-focused design did not negatively impact the ability to image ex vivo lumpectomies. Due to the rigid design of the device, imaging the lateral surfaces of surgical cavities required aiming the device’s screen away from the user. An external display onto which the images from the handheld device could be projected would rectify this limitation if placed across from the patient and directly in the user’s line of sight.

The Eagle device’s aluminum shaft, designed to reach into and image surgical cavities, has diameter 24 mm and length 91 mm. The smallest cavity dimensions measured were $2\times 3\times {1.5}\,\text {cm}$ ($l\times w\times d$). The imaging tip’s diameter was sufficiently small to reach inside these cavities, although this size is near the acceptable diameter limit. Maximum cavity depth was measured to be 9 cm. The length of the imaging shaft is sufficient to image cavities beyond this length—considering the 2 cm minimum autofocus distance, cavities with depths of up to approximately 11.1 cm can be imaged with the maximum possible resolution. Cavities with larger depths may be imaged but with a larger imaging distance (>2 cm) and thus reduced resolution. In the future, further miniaturization of the distal tip (optics) could be considered in order to expand the indications for this type of handheld fluorescence imaging device.

The Eagle device’s 8 MP image sensor can resolve 89 $\upmu$m from 3 cm. This is sufficient to visualize a small cluster of FL tumour cells. A higher resolution image sensor may be capable of visualizing PpIX FL produced by a single cancerous cell. However, the luminance of the FL produced by a single cell may not be strong enough to overcome the luminance from nearby healthy tissues. Improved PpIX detection may result from modifications to the imaging filter. Expanding the red band to approximately 725 nm would increase the PpIX FL transmitted to the camera (see Fig. 1a). Since green connective tissues are often the brightest regions in images captured of breast tissues with the Eagle FL device, decreasing the transmission of the green band of the filter may further improve PpIX-to-normal contrast, enabling the PpIX luminance to better stand out among healthy tissues. However, since PpIX is a naturally-occurring molecule in healthy cells, and oral exogenous 5-ALA increases the production of PpIX systemically, amplifying the PpIX-to-normal contrast may lead to false positives.

A potential source of false negatives is due to PpIX photobleaching. Users were trained to minimize tissue light exposure during imaging to avoid photobleaching PpIX before image capture. Stray excitation light outside of the FOV may photobleach PpIX outside the FOV before imaging, so imaging with speed is critical. The Eagle prototype’s imaging speed may be improved to minimize the probability of photobleaching. In the trial, videos of the surgical cavity were captured, followed by image capture of the posterior margin. Implementing an image capture function during video recording would eliminate the necessity of scanning a single area multiple times. Additionally, image capture time currently takes 1-2 s, which increases the risk of blurry images due to the user’s movements and increases the need for additional images of the same region to be taken. Furthermore, the area of the cropped FOV is only 46% of the maximum possible area based on the camera’s angle of view $\theta$. The shrunken FOV resulted in cavity scans taking longer than the desired 1-2 min. Reducing the image-capture time, combining WL and FL capabilities into a single device, implementing image capture during video recording, and increasing the FOV will reduce the overall imaging time.

Draping the device was also a time-consuming procedure. A drape that is easier to install and remove would simplify the draping process, increase the rate of successful initial draping without compromising sterility, and decrease the time required to prepare the Eagle device for imaging within the sterile field of the OR.

The drape was also observed to interfere with the touchscreen display. The drape produced reflections in lit rooms, which made viewing the screen difficult. An external display, as suggested previously, may mitigate this. Secondly, the drape added another layer between the user’s finger and the touchscreen (in addition to gloves), decreasing the screen sensitivity. Redundant physical buttons to control critical functions during draped imaging may improve usability.

### Suggested mitigation of imaging artifacts

In this clinical study, several imaging artifacts were discovered: surgical drape-induced artifacts, illumination and FOV artifacts, and blood in the surgical cavity. Device modifications may mitigate these artifacts.

The surgical drape was the source of several imaging artifacts. When dirty, the drape’s window produced a green tint across images. Cleaning the window with lens cleaning spray and lens paper reduced the tint. However, cleaning a dirty drape did not reduce the tint to the same level as in Fig. 3d (i.e., with a newly opened, clean drape). Cleaning the drape’s window is advisable when fingerprints are present, but careful handling of the drape to avoid debris on the optical window is preferred. Furthermore, spraying the drape window with lens cleaner cannot be done in a sterile surgical field without compromising the sterility of the drape. Although the colour contrast is higher in images captured with a dirty drape, image quality is sacrificed. Surfaces with low luminance under FL imaging (e.g. adipose tissues) are most susceptible to hue changes [45]. Therefore, when a source of green tint is present, adipose tissues and other low-luminance ROIs appear green, which decreases adipose-connective contrast, thereby decreasing the ability to differentiate these tissues. Generally, according to data collected with the Eagle device and presented in this manuscript, aiming for $\Delta E\gg 50$ is unnecessary. Achieving contrast of $\Delta E\approx 50$ by imaging with a clean drape and replacing fluorescent materials in the window with non-fluorescent substitutes will not create greater difficulty differentiating two colours.

The drape’s window was also observed to accumulate condensation while imaging the surgical cavity. The source of the condensation is hypothesized to be the patient’s body heat and humidity in the surgical cavity. As imaging progressed and the temperature of the device tip increased due to the heat produced by the 405 nm LEDs, the temperature difference between the surgical cavity and drape window decreased, eliminating the buildup of the condensation. Therefore, heating the device tip before imaging by turning on the LEDs to initially decrease the temperature difference between the cavity and the imaging tip of the device may reduce the condensation. Preheating the LEDs for $\sim$2 min (the average imaging time required before condensation buildup was no longer observed) immediately before imaging may be sufficient to eliminate the haze when imaging.

The Eagle device’s illumination uniformity was deemed suboptimal due to the relative placement of the LEDs and the camera. Specifically, illumination was focussed near the bottom of the FOV. Better uniformity across the FOV would aid in visualizing off-centre FOV regions. The uniformity may be improved by placing LEDs on all sides of the camera rather than focussing them on the bottom. By arranging the blue and white LEDs on the sides of the camera, it could allow both imaging modes to be performed with one device in future versions. Additionally, including a circle directly on the user interface, such as the one drawn in Fig. 6c, may help the user ensure their imaging ROI is centred and within the region of maximum uniformity before image capture.

Blood was observed to be potentially similar in appearance to PpIX. Removal of pooled blood, as commonly done during BCS, would help eliminate artifacts due to blood in the surgical cavity. WL imaging may be used before FL to determine whether the imaging field is free of blood. In addition, adding excitation filters to the 405 nm LEDs would decrease the quantity of red light emitted from the LEDs reflecting off the blood and give it a darker appearance with greater PpIX contrast. Excitation filters would also reduce or remove the reflections consistently observed from the 405 nm LEDs on glossy or liquid surfaces, as shown in Fig. 5c and d, for example (these reflections are likely due to light emitted by the LEDs within the 500 to 550 nm range of the imaging filter’s green band).

## Conclusions

This pilot study has demonstrated the detection of a grossly occult positive margin intraoperatively. We were also able to distinguish healthy breast tissues based on their appearance and demonstrated the detection of red FL tumours in sectioned lumpectomies following administration of 5-ALA. Imaging breast surgical cavities for the first time with the Eagle device has elucidated the strengths and weaknesses of the prototype device. Artifacts from imaging within the surgical cavity were identified, and potential mitigations have been proposed.

Since discovering these imaging artifacts, SBI ALApharma Canada (Toronto, Canada), who has licensed the intellectual property relating to this technology from MolecuLight, has developed a second iteration of the Eagle imaging platform to evaluate many of the proposed mitigation solutions and the subsequent impact on intraoperative detection of BCS positive margins.

## Data Availability

No datasets were generated or analysed during the current study.

## Abbrevations

5-ALA: 5-aminolevulinic acid
AOI: Angle of incidence
BCS: Breast conserving surgery
BW: Bodyweight
CIE: International commission on illumination
CMOS: Complementary metal oxide semiconductor
DCIS: Ductal carcinoma in situ
DMSO: Dimethyl sulfoxide
FDA: U.S. food and drug administration
FL: Fluorescence
FOV: Field of view
FWHM: Full width at half maximum
H[MYAMP: E] Hematoxylin and eosin
HCl: Hydrochloride
IDC: Invasive ductal carcinoma
LCD: Liquid crystal display
LED: Light-emitting diode
MSH: Mount Sinai hospital; Toronto, Canada
OD: Optical density
OLED: Organic LED
OR: Operating room
PA: Pathologists’ assistant
PMCC: Princess Margaret cancer centre; Toronto, Canada
PpIX: Protoporphyrin IX
PRODIGI: Portable real-time optical detection, identification and guide for intervention
RCT: Randomized controlled trial
REB: Research ethics board
ROI: Region of interest
SSO-ASTRO: Society of surgical oncology-american society for radiation oncology
UHN: University health network; Toronto, Canada
WL: White light
